# The Free‐Falling Needle: A Case Report of an Accidental Iatrogenic Pneumothorax

**DOI:** 10.1002/ccr3.72285

**Published:** 2026-04-24

**Authors:** Abdul Qadir, Amal Wael Abdellatif, Elmukhtar Habas, Mamunul Islam

**Affiliations:** ^1^ Department of Medicine Hamad Medical Corporation, HGH Doha Qatar; ^2^ Medical Education Department Hamad Medical Corporation Doha Qatar

**Keywords:** accidental needle drop, breast cyst aspiration, chest radiograph, conservative management, iatrogenic pneumothorax, patient safety

## Abstract

This case highlights the potential for accidental iatrogenic pneumothorax during routine procedures, like breast cyst aspiration, and emphasizes the importance of strict procedural precautions to avoid such incidents.

## Key Clinical Message

A needle accidentally dropped onto a patient's chest during breast cyst aspiration caused a small iatrogenic pneumothorax—a novel and previously unreported mechanism. This case underscores that even routine, minimally invasive procedures demand strict operator focus and needle discipline to prevent unintended, potentially serious thoracic injury.

## 1 Introduction

Pneumothorax is a clinical entity that is bound to appear in an internist's practice. It happens when injury to the lung leads to the efflux of air into the potential space between the visceral and parietal pleura. Pneumothorax arises in patients with different respiratory risk profiles; hence, the wide range of severity of individual patient presentations.

Primary pneumothorax occurs in the absence of any lung pathology and is attributed to a sub‐pleural air bleb rupture. Primary pneumothorax is usually self‐limited and not associated with severe respiratory impairment [1]. However, secondary pneumothorax (SP) occurs in patients with pre‐existing lung diseases like chronic obstructive pulmonary disease (COPD) or cystic fibrosis. Due to lower pulmonary reserve in SP patients, they usually have a more severe presentation and require more urgent interventions. Iatrogenic pneumothorax occurs secondary to a diagnostic or a therapeutic procedure. The mechanism of injury in iatrogenic pneumothorax—intentional versus accidental—has significant implications with regard to patient safety.

Invasive procedures like transbronchial biopsy and thoracic surgeries entail intentional, direct puncturing of the lung and therefore carry an intrinsic risk of causing pneumothorax [2]. In contrast, other less‐invasive procedures like thoracentesis, central venous catheterization, and breast biopsy are the ones in which the term “accidental” is ascribed to the lung injury [2].

Such procedures are the ones in which safety precautions on the part of the operating doctor are called into question since exercising caution in those scenarios can reduce the incidence of iatrogenic pneumothorax.

A study that investigated over 3900 patients admitted for lung, cardiac, vascular, and other procedures known to carry a risk for pneumothorax revealed an overall incidence rate of 0.67 per 1000 discharges at risk of developing Accidental Iatrogenic Pneumothorax (AIP) [2].

Table 1 lists the most common surgeries/procedures implicated in AIP in the study in *descending* order of incidence rate. The listed culprit procedures and their matching incidence rates closely resemble those of another study that investigated 164 patients diagnosed with AIP [3], with central venous catheterization and thoracentesis being the top causes. AIP tends to increase the cost of health care, prolong the affected patients' hospital stay, and increase their morbidity and mortality [2].

**TABLE 1:** The common causes of AIP and their incidence.

Procedure/surgery	Proportion of all IAP cases
Central venous catheterization	26.5%
Thoracentesis	24%
Mechanical ventilation	21%
Cardiac pacemaker or ICD‐related procedures	12%
Respiratory procedures outside OT	7.7%
Bronchoscopy and bronchial biopsy	7.5%
Tracheostomy	6.8%
Skin and breast procedures outside OT	5%
Gastrostomy	4.7%
Nephrectomy	3%

Abbreviations: ICD: implantable cardioverter defibrillator; OT: operating theater.

In this report, we present an interesting case of Iatrogenic pneumothorax. To our knowledge, this is the first case report documenting AIP secondary to the accidental drop of a needle on a patient's chest wall.

## 2 Case History/Examination

A 35‐year‐old patient presented to the emergency department with right‐sided chest pain. Around 6 h earlier, the patient was in the clinic for breast cyst aspiration when an anesthesia needle had fallen on her chest.

The patient, who has a history of bilateral breast lesions, was scheduled for ultrasound‐guided core biopsy and aspiration of her left breast mass and right breast cyst, respectively.

As the operating doctor was handling the anesthesia needle, the doctor shifted his/her gaze away from the patient. As a result, the doctor inadvertently dropped the needle, pricking the superomedial aspect of the patient's right breast. The patient immediately started having sharp chest pain. The pain was worse with inspiration; however, she was vitally stable with an oxygen saturation of 99% on room air. After aspirating fluid from the right breast cyst and concluding the procedure, the patient still had significant pain. Accordingly, the patient was transferred to the ED to be evaluated for pneumothorax.

In the ED, the patient was afebrile and had an oxygen saturation of 99% on room air. Blood pressure was 124/71 mmHg, respiratory rate 16, and peripheral pulse rate 68/min. She reported having sharp, right‐sided chest pain that was worse with deep inspiration and mild shortness of breath (SOB). She also reported a “crackling noise” that she appreciated upon bowing down. On physical exam, the patient appeared well but mildly anxious. Lung auscultation revealed clear, equal air entry bilaterally. There was no hyperresonance to percussion on anterior and posterior lung fields. No crepitus was appreciated upon superficial palpation of the chest wall. Heart auscultation revealed normal S1 and S2 sounds without murmurs.

## 3 Differential Diagnosis, Investigations and Treatment

**FIGURE 1:**
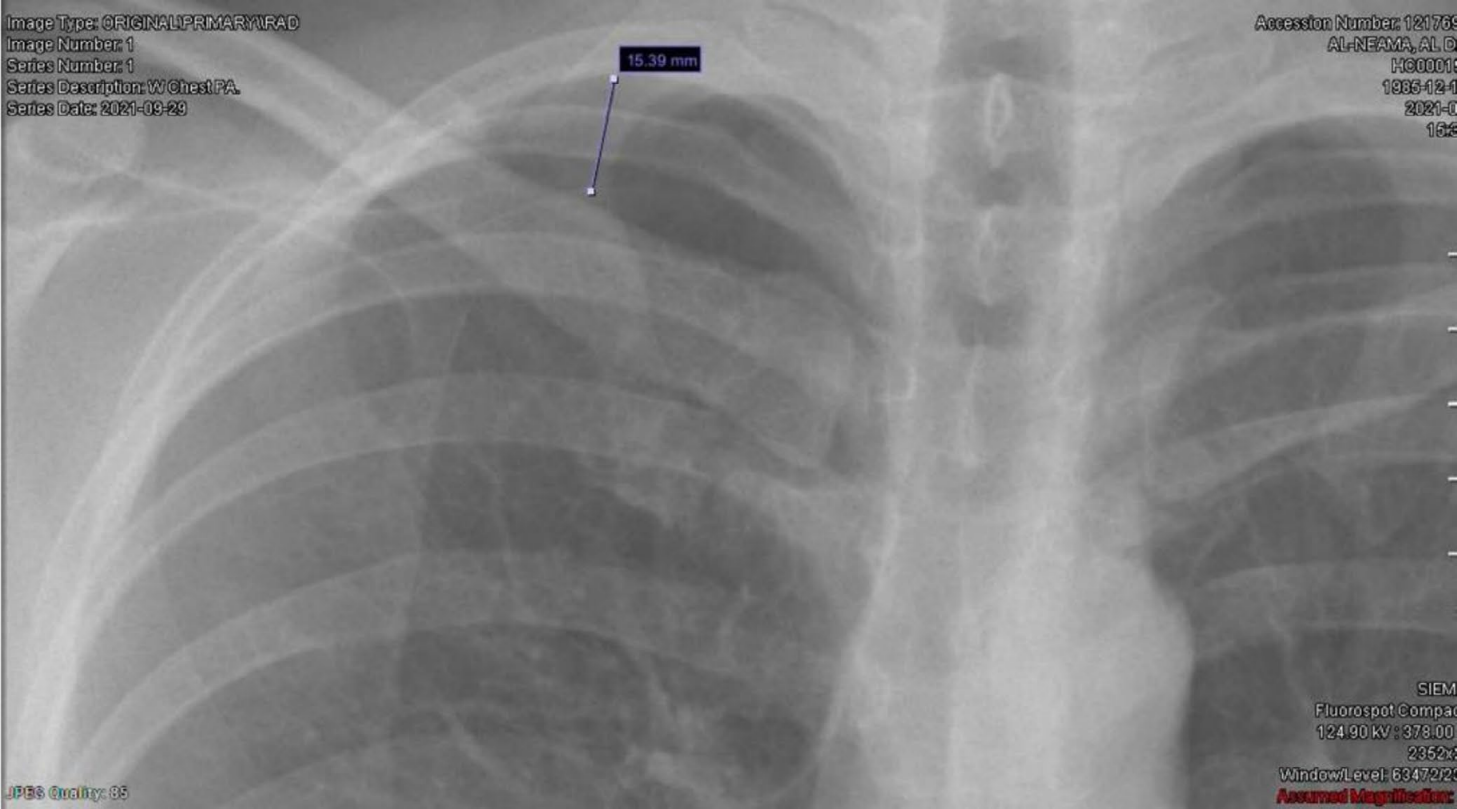
Chest X‐ray showing a 1.5 cm right‐sided apical pneumothorax.

Her chest x‐ray (CXR) showed a 1.5 cm, right‐sided apical pneumothorax measured from the lung apex to the cupula (cervical segment of the parietal pleura) (Figure 1). The thoracic surgeon on‐call was consulted, and he recommended conservative management and a repeat CXR the next day to reassess the size of the pneumothorax. Accordingly, the patient was admitted for observation, given Ketorolac for pain relief, and started on 2 L of nasal cannula.

## 4 Conclusion and Results (Outcome and Follow‐Up)

**FIGURE 2:**
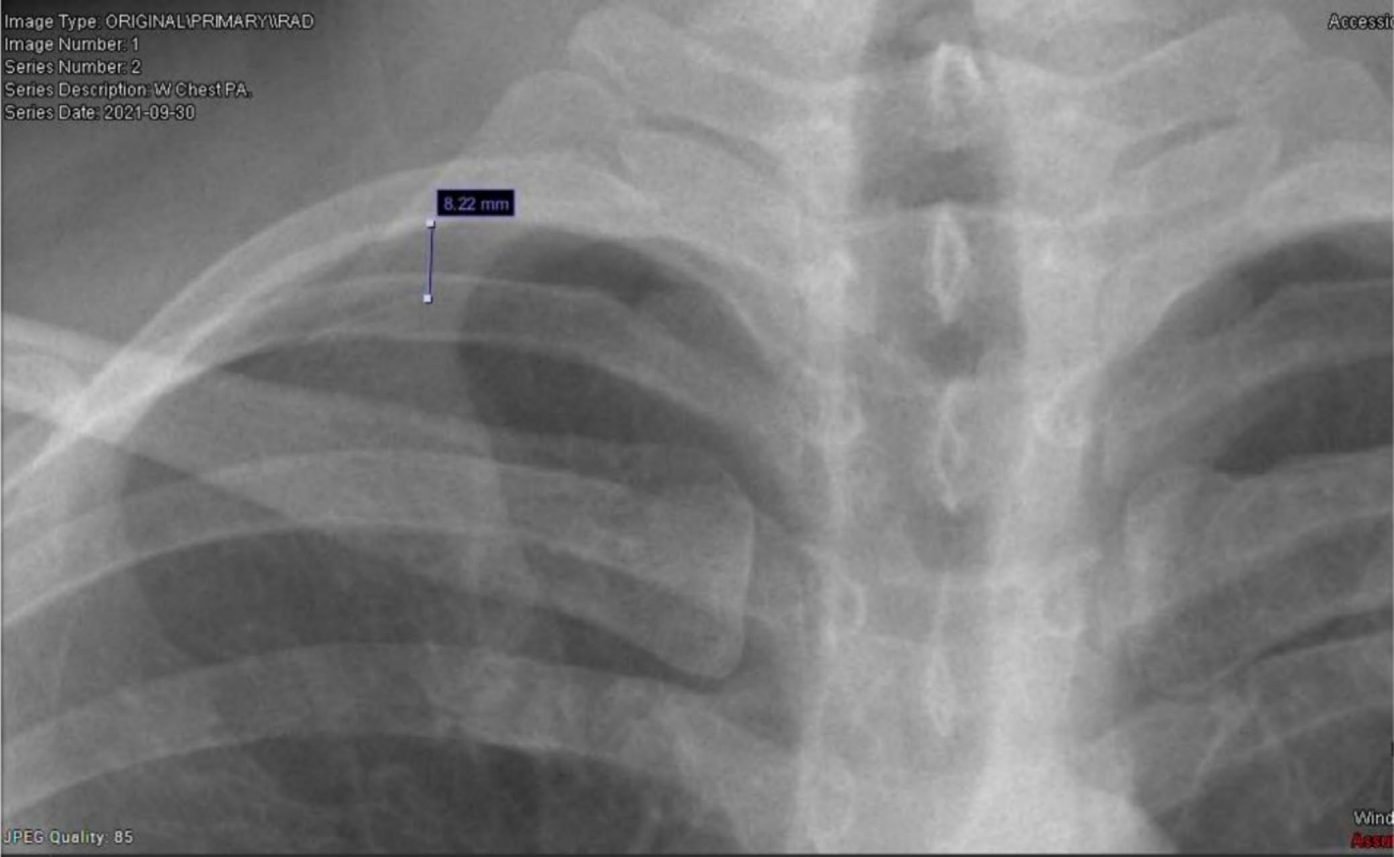
Follow‐up chest X‐ray showing regression of the right‐sided apical pneumothorax to 0.8 cm after conservative management.

Repeat CXR on the following day revealed that the pneumothorax had regressed to 0.8 cm (Figure 2). A decision was made to discharge the patient with a follow‐up appointment within one week in order to undergo a chest CT scan. Upon discharge, the patient was comfortable, saturating at 99% on room air, and not complaining of chest pain or SOB. A week later, a chest CT scan revealed no evidence of pneumothorax or underlying lung pathology potentially implicated in SP.

## 5 Discussion

Our patient– a young, healthy woman without an underlying lung disease– developed iatrogenic pneumothorax due to an accidental drop of an anesthesia needle. Owing to her small pneumothorax (< 3 cm) and her very mild symptoms, she was treated conservatively as recommended by the American College of Chest Physicians (ACCP) [1] consensus statement. Although she was offered a follow‐up chest CT, the ACCP does not recommend such practice for patients with a first‐time pneumothorax without clinical evidence of an underlying lung disease [1].

We believe our patient's case merits discussion since its incidence is highly unexpected in a state‐of‐the‐art clinical setting where a trained doctor performed the procedure. The case highlights the importance of taking strict precautions while performing relatively simple procedures like breast biopsy or cyst aspiration. The accident that happened to our patient could have been avoided had the operating doctor not held the needle above the breast while she was not using it. Optimum care and focus are expected from well‐trained doctors while handling any sharp objects close to their patients.

Our literature review revealed no previous incidence of a similar case, probably due to poor reporting in fear of malpractice lawsuits. We hope that by reporting such a case, we can (a) alert physicians to the possibility of the occurrence of a similar scenario and (b) prompt them to exercise more caution to minimize the risk of unintended injury.

## Author Contributions

**Abdul Qadir:** conceptualization, data curation, formal analysis, investigation, methodology, writing – original draft, writing – review and editing. **Amal Wael Abdellatif:** conceptualization, formal analysis, funding acquisition, investigation, methodology, writing – original draft, writing – review and editing. **Mamunul Islam:** formal analysis, investigation, project administration, writing – review and editing. **Elmukhtar Habas:** conceptualization, methodology, validation, visualization, writing – review and editing.

## Funding

The authors have nothing to report.

## Ethics Statement

The Medical Research Center at Hamad Medical Corporation in Qatar has granted approval for the publication of this case report.

## Consent

Written informed consent was obtained from the patient for publication of the details of their medical case and any accompanying images.

## Conflicts of Interest

The authors declare no conflicts of interest.

## Data Availability

The data that support the finding of this case report are contained within the article. Additional information is available from the corresponding author upon reasonable request and with the approval of the relevant ethics committee.

## References

[1] M. H. Baumann , C. Strange , J. E. Heffner , et al., “Management of Spontaneous Pneumothorax: An American College of Chest Physicians Delphi Consensus Statement,” Chest 119, no. 2 (2001): 590–602. 11171742 10.1378/chest.119.2.590

[2] C. Zhan , M. Smith , and D. Stryer , “Accidental Iatrogenic Pneumothorax in Hospitalized Patients,” Medical Care 44, no. 2 (2006): 182–186. 16434918 10.1097/01.mlr.0000196938.91369.2a

[3] B. Celik , E. Sahin , A. Nadir , and M. Kaptanoglu , “Iatrogenic Pneumothorax: Etiology, Incidence and Risk Factors,” Thoracic and Cardiovascular Surgeon 57, no. 5 (2009): 286–290. 19629891 10.1055/s-0029-1185365

